# Massive systemic arterial air embolism after computed tomography-guided lung biopsy: a rare cardiovascular complication—case report

**DOI:** 10.1093/ehjcr/ytag120

**Published:** 2026-02-14

**Authors:** Emídio Mata, Bárbara Lage Garcia, Sara Borges, Frederico Cavalheiro

**Affiliations:** Cardiology Department, Unidade Local de Saúde do Alto Ave, R. dos Cutileiros 114, Creixomil, 4835-044 Guimarães, Braga, Portugal; Cardiology Department, Unidade Local de Saúde do Alto Ave, R. dos Cutileiros 114, Creixomil, 4835-044 Guimarães, Braga, Portugal; Cardiology Department, Unidade Local de Saúde do Alto Ave, R. dos Cutileiros 114, Creixomil, 4835-044 Guimarães, Braga, Portugal; Radiology Department, Unidade Local de Saúde do Alto Ave, R. dos Cutileiros 114, Creixomil, 4835-044 Guimarães, Braga, Portugal

## Summary

This case highlights a rare but catastrophic complication of lung percutaneous computed tomography (CT)-guided transthoracic needle biopsy (LPCNB) characterized by the simultaneous disruption of an air-containing space (such as alveoli) and a nearby pulmonary vein, creating a temporary fistulous communication that in moments of increased intrathoracic pressure (such as coughing) forces air into the bloodstream.^1,2^

## Case description

A 69-year-old white male with moderately differentiated rectal adenocarcinoma, previously treated with radiotherapy and surgical resection, was found on follow-up to have a 9 mm hypermetabolic nodule in the right middle lung lobe, suspicious for synchronous pulmonary malignancy. He was referred to LPCNB. He was functionally independent, with a history of hypertension, dyslipidaemia, ischaemic heart disease treated with stent, and active smoking. Baseline laboratory values were within normal limits, physical examination was unremarkable, and ECG showed sinus rhythm without acute changes.

**Figure 1:**
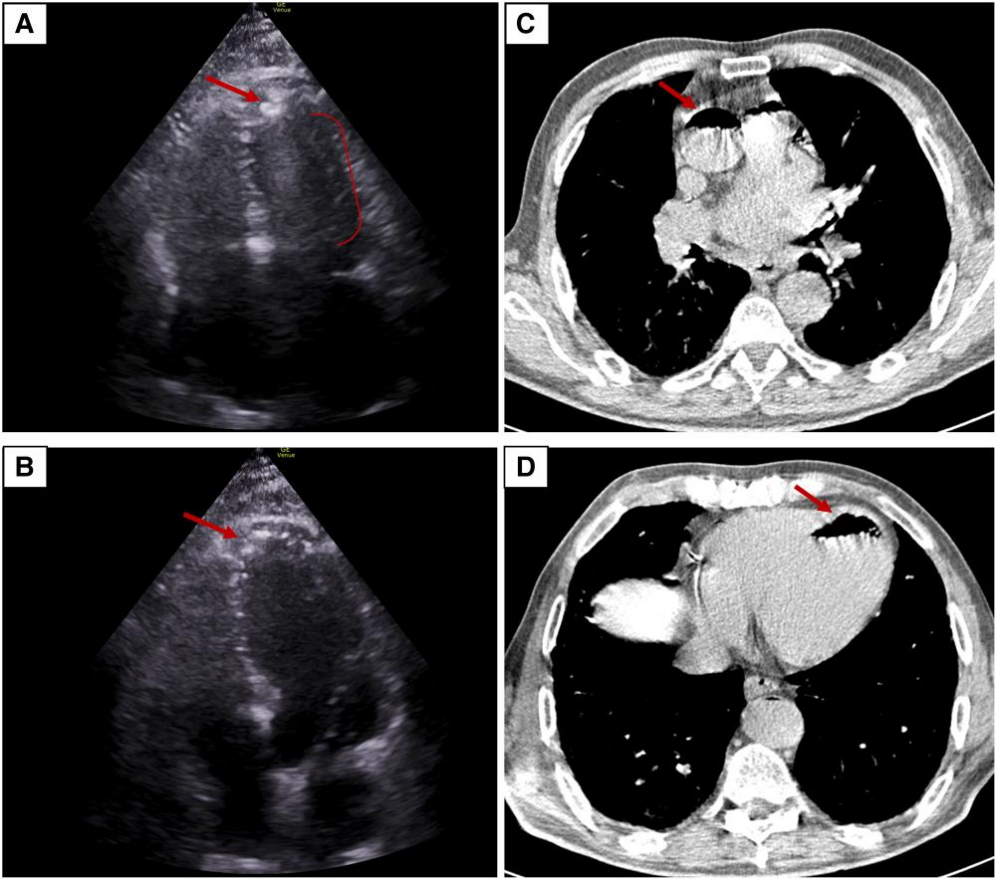
Systemic arterial air embolism following percutaneous lung biopsy. Following sudden-onset coughing and cardiac arrest during lung biopsy, transthoracic echocardiography (*A, B*) revealed air bubbles in the left ventricle (arrow), associated with a dynamic, cone-shaped artefact synchronous with the cardiac cycle. Chest computed tomography confirmed the presence of air (arrows) within the ascending aorta (*C*) and left ventricle (*D*).

During the procedure, the patient developed sudden-onset coughing, followed by profuse haemoptysis and acute dyspnoea. Shortly thereafter, he became unresponsive and experienced cardiac arrest. Cardiopulmonary resuscitation was promptly initiated for pulseless electrical activity, with return of spontaneous circulation achieved after one cycle. Transthoracic echocardiography acquired a rare image of air bubbles in the left ventricle, accompanied by a cone-shaped artefact (*Figure 1A* and *B* and Supplementary  *Video*). These findings prompted immediate placement of the patient in the Trendelenburg position.

Subsequent chest CT confirmed the presence of air within the left ventricle and the aorta (*Figure 1C* and *D*), along with a minimal anterolateral pneumothorax. Unfortunately, brain CT revealed hypodense cortical–subcortical areas in the left frontal region, consistent with acute ischaemic changes secondary to air embolism.

The patient was urgently transferred for hyperbaric oxygen therapy, and after a 6-h session, repeat CT showed resolution of intravascular air, while retaining the ischaemic brain lesions.

Upon admission, the patient developed generalized clonic movements, initially suppressed with higher propofol infusion. Electroencephalogram confirmed convulsive status epilepticus with left frontotemporal onset and rapid bilateralization. Despite sequential therapy with levetiracetam, phenytoin, and phenobarbital, seizures persisted. A multidisciplinary decision was made to attempt a single cycle of burst-suppression with propofol and midazolam, but epileptiform discharges continued, and repeat electroencephalogram confirmed refractory status epilepticus. Supportive measures, including targeted temperature control and metabolic optimization, were maintained, yet no neurological improvement was observed.

Given the extent of cerebral injury and poor neurological prognosis, the multidisciplinary team faced the challenge of communicating futility of further invasive measures to the patient’s family. Through structured, empathetic discussions with his close relatives, consensus was reached to withhold further life-sustaining interventions. The patient died shortly thereafter.

## Discussion

Large series have estimated the incidence of LPCNB-related air embolism between 0.02 and 0.4%, with outcomes ranging from transient neurological deficits to sudden death.^3^ In the multicentre survey by Tomiyama *et al*., 6 of 9783 biopsies (0.06%) were complicated by systemic air embolism,^4^ while Hiraki *et al*. and Ibukuro *et al*. reported risks of 0.4% and 0.2%, respectively.^5,6^ Among 19 published cases summarized by Ibukuro *et al*., over half of the patients developed neurological sequelae, more than a third suffered cardiac complications, and one quarter died. Recognition of this complication is challenging, as some events may be clinically silent. Immediate management consists of Trendelenburg positioning and hyperbaric oxygen therapy, which can be life-saving when initiated promptly.^3^

## Lead author biography


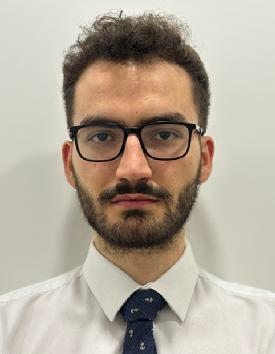


Emidio Mata is a Cardiology Resident at Unidade Local de Saúde do Alto Ave, Portugal. He completed his Integrated Master’s in Medicine at Universidade do Minho and began his residency following a year of general training. His research interests include electrophysiology, structural heart disease, cardio-oncology, and public health. Dr Mata has authored several peer-reviewed publications and actively contributes as a peer reviewer, including for the *Journal of the American College of Cardiology*. He has a strong foundation in clinical data analysis and medical informatics, supporting an evidence-based and forward-looking approach to cardiovascular medicine.

## Supplementary Material

ytag120_Supplementary_Data

## Data Availability

All relevant data, including the cardiology-related images, are fully displayed and described within the manuscript. No additional datasets were generated or analysed for this article.

## Supplementary material

**Funding:** None declared.
